# Exploring the Cross-cultural Applicability of a Brief Compassionate Mind Training: a Study Comparing Sri Lankan and UK People

**DOI:** 10.1007/s12671-022-02041-z

**Published:** 2022-12-23

**Authors:** Lasara Kariyawasam, Margarita Ononaiye, Chris Irons, Sarah E. Kirby

**Affiliations:** 1 University of Southampton, Highfield Campus, University Road, Southampton, SO17 1BJ UK; 2 University of Southampton, Southampton, UK; 3 Balanced Minds, Edinburgh, Scotland; 4 Academic Unit of Psychology, University of Southampton, Southampton, UK

**Keywords:** Compassion, CMT, Efficacy, Sri Lankan, UK, Cross-cultural

## Abstract

**Objectives:**

Compassionate Mind Training (CMT) is a therapeutic approach proven to be effective for reducing distress and increasing well-being in clinical and non-clinical populations. This study aimed to explore the efficacy of a short-term, online version of the CMT on compassion, distress, and well-being in a cross-cultural, non-clinical sample of Sri Lankan and UK people.

**Method:**

A randomized controlled trial with pre-, post-measurements, and a 2-week follow-up was conducted using CMT (*n* = 21 Sri Lankan, *n* = 73 UK) and wait-list control (*n* = 17 Sri Lankan, *n* = 54 UK) groups. The intervention effects were investigated using a series of repeated-measures ANOVAs using intention-to-treat and per-protocol analyses.

**Results:**

The 2-week CMT was effective in increasing all aspects of compassion in both Sri Lankan and UK people. In addition, some cross-cultural similarities and differences (in the factors affecting compassion) were present in the improvements following CMT between the two countries, which were maintained at a 2-week follow-up.

**Conclusion:**

This study provides promising evidence for the efficacy and cross-cultural applicability of CMT for reducing distress and increasing well-being.

Compassion has received an increased interest in research and psychotherapy over the last two decades (Kirby, 2016; Matos et al., 2017a). Practicing compassion has been found to produce various physiological (Fredrickson et al., 2013), psychological (e.g., Keltner et al., 2014), and social benefits (Crocker & Canvello, 2012). In fact, studies have found that compassion is linked to several factors such as coping with distress and failures (Leary et al., 2007), decreased anger, anxiety, shame (Barnard & Curry, 2011), and self-criticism (Neff, 2003), and increased positive affect, optimism, and happiness (Neff et al., 2007). Therefore, several compassion-based interventions have been introduced, aiming to reduce distress and increase well-being in clinical and non-clinical populations (Neff & Germer, 2013).

One such intervention is Compassion-Focused Therapy (CFT: Gilbert, 2000, 2010a), which was originally developed as a psychotherapy for patients with high shame and self-criticism. CFT attempts to cultivate care-based motives, intents, and soothing affiliations, and alleviate persistent patterns of distress, to help people combat shame-based and traumatic experiences (Gilbert, 2020; Irons & Heriot-Maitland, 2021; Kirby, 2016). Compassionate Mind Training (CMT) is integrated in CFT and entails several practices to facilitate compassion and the psychoeducation that compassion is a sensitivity to suffering of oneself and others with a commitment to try to relieve and prevent suffering (Gilbert, 2017a).

Gilbert (2014), one of the leading theorists in this area, emphasized that emotions are evolved to serve certain functions that are clustered in a system known as the *affective regulatory system*, which contains three interactive systems: *threat*, *drive*, and *soothing*. He posits that cultivating the soothing system through practicing compassion for oneself and others may be helpful in regulating distress. Gilbert emphasized that compassion can be experienced across three directional flows—*self-compassion*, *compassion towards others*, and *compassion from others*—and these compassionate experiences are often challenged by fears, blocks, and resistances (Irons & Heriot-Maitland, 2021).

To cultivate compassion and well-being by reducing the impact of fears, blocks, and resistances, CMT provides psychoeducation that much of the suffering which humans experience is beyond their control and, therefore, is not their fault (Gilbert, 2009, 2014). CMT comprises a series of practices designed to enhance sociality, friendliness, mindfulness, and well-being, particularly among people with high shame and self-criticism (Irons & Heriot-Maitland, 2021; Kirby, 2016; Matos et al., 2017a). These practices include physiological processes such as breathing (e.g., soothing rhythm breathing), imagery (e.g., safe space imagery), body posture, and voice tone training designed to facilitate self-awareness, self-grounding, and a sense of a compassionate self (Matos et al., 2017a).

By promoting self-awareness, CMT also increases mindfulness, which is the ability to pay attention to the present moment without the interference of judgment while acknowledging the distractions (Gilbert, 2010; Irons & Heriot-Maitland, 2021). This allows individuals to refocus when the mind wanders and gets distracted from the present moment. Mindfulness also facilitates CMT in turn, by allowing people to engage in CMT practices without being distracted and, when distracted, by allowing them to refocus. For instance, practicing CMT exercises such as soothing rhythm breathing or imagery could be difficult for a beginner, as their attention could be easily distracted by various thoughts and cognitions. Developing the ability to mindfully focus on the breathing or imagery would tackle the distracting thoughts and gently bring one’s focus back to the CMT practices (Gilbert, 2010b).

Studies have found that the embodiment of a compassionate self has increased optimism (Meevissen et al., 2011), coping behaviors (Peters et al., 2010), and mood (Osimo et al., 2015), and the activation of the soothing system via practicing CMT has increased well-being and prosocial motivations for self and others (Kirby et al., 2017). Interestingly, the predominance of the research has focused on self-compassion (e.g., Arimitsu, 2016; Wong & Mak, 2016), with only a few studies exploring compassion across the three flows (Irons & Heriot-Maitland, 2021), highlighting the need for future research.

In addition, CMT has been found to increase other factors such as self-reassurance, social safeness, and pleasure (Irons & Heriot-Maitland, 2021; Maratos et al., 2019, 2020), which are known as facilitators of compassion (Gilbert, 2017b). On the other hand, in addition to reducing psychopathology such as anxiety and depression (e.g., Matos et al., 2022a, 2022b), CMT has been found to reduce fears of experiencing compassion, self-criticism, and external shame, such as the perception that others in the society judge and criticize oneself (e.g., Gilbert & Procter, 2006; Irons & Heriot-Maitland, 2021). These factors (fears, self-criticism, and external shame) are frequently considered inhibitors of compassion due to their negative correlation with compassion (Gilbert, 2017b). In line with this, a cross-cultural comparison between Sri Lankan and UK people found that although Sri Lankans reported higher self-compassion and self-reassurance, they indicated higher external shame and fears of compassion for the self and others (Kariyawasam et al., 2022a, 2022b). In comparison, UK participants reported significantly higher levels of social safeness and pleasure and were not fearful of engaging compassionately with others. CMT appears to increase facilitators of compassion and reduce inhibitors of compassion, in both clinical (Beaumont & Martin, 2016; Gilbert & Procter, 2006) and non-clinical populations (Matos et al., 2017b).

Despite the trans-diagnostic and multifaceted nature of CMT (Matos et al., 2017a), most of the research has been conducted in Western countries (Halamova et al., 2020). This is surprising given that almost all compassion-based interventions including CFT and CMT are influenced by Buddhist philosophies that are embraced across a range of predominantly Asian cultures (Kirby, 2016). Despite this, until recently, there has been a lack of research attempting to enhance compassion in Buddhist-influenced Asian cultures such as Japan (Arimitsu, 2016) and Sri Lanka (Kariyawasam et al., 2021, 2022a). In fact, a recent meta-analysis concluded that the limited existing compassion-based studies in Asian countries have been conducted within the last 5 years (Kariyawasam et al., 2022b). Despite the scarcity of research, existing literature indicates that compassion-based interventions can increase well-being and reduce shame and criticism in Asian cultures (e.g., Arimitsu, 2016), highlighting the need for further research.

This study therefore aimed to investigate the efficacy of a 2-week online CMT in a cross-cultural group of Sri Lankan and UK people. This was the first study to explore CMT in an Asian sample in comparison to a Western sample. Online CMT studies are distinctly scarce (Halamova et al., 2020) and there is also a dearth of cross-cultural CMT studies (Maratos et al., 2019, 2020; Matos et al., 2021, 2022a). Additionally, the use of an online CMT was particularly appropriate due to the current climate of the COVID-19 pandemic for both Sri Lankan and UK participants (Halder, 2020; Wang et al., 2020). Therefore, the aim of this study was to explore the impact of CMT on the three flows of compassion and the inhibitors and facilitators of compassion (Gilbert, 2014).

The research aimed to answer the research question: whether CMT will increase the three flows of compassion in the CMT group when compared to the wait-list control group, regardless of the cultural background. This study explored whether CMT will improve the facilitators (self-reassurance, social safeness and pleasure, and well-being) and decrease the inhibitors of compassion (fears of compassion, self-criticism, external shame, anxiety, and depression). In addition, any cross-cultural differences across these factors between Sri Lankan and UK people were investigated. If there are any changes, this study also explored whether these will be maintained at a 2-week follow-up.

## Method

### Participants

Participants were recruited purposively via social media and universities from Sri Lanka and the UK. The first 40 Sri Lankan university students to complete the entire study received a £5 Amazon voucher each, and UK university students received course credit for their participation. All participants self-identified as Sri Lankan or UK nationals, were aged at least 18 years or older, and were able to understand spoken and written English.

Overall, 477 participants (232 SL and 245 UK) signed up for the study and completed the baseline-1 (T1) measures. In the Sri Lankan sample, 119 participants were in the CMT (Compassionate Mind training) group, and 113 participants were in the WLC (Wait-List Control) group. In the UK sample, 125 participants were in the CMT group, and 120 participants were in the WLC group. However, only 21 (17.6%) in the Sri Lankan CMT group and only 73 (58%) in UK CMT group completed the post-CMT measures at T2. In the WLC groups, only 17 In the Sri Lankan group and only 54 in the UK group completed T2. Indicating a further attrition rate, only 19 (15.9%) in the Sri Lankan CMT group and 36 (28.8%) in the UK CMT group completed the follow-up measures at T3.

### Procedures

This study used an online, randomized controlled trial (RCT) with a pre-post and 2-week follow-up design in a cross-cultural group of Sri Lankan and UK participants. The entire study was 1 month in duration, including a 2-week CMT and a follow-up test. A study advert was published on social media and on university platforms (such as noticeboards, student emails as approved, and emailed by the universities). Those who indicated interest by signing up or emailing the researchers were emailed a link which contained an information sheet and consent form along with a series of questionnaires. Participants could only access the questionnaires after indicating consent to participate. This study was hosted on the Qualtrics online survey platform.

The questionnaires included a demographic form, CEAS, FOCS, FSCRS, OAS, SSPS, GAD-7, WEMWBS, and the practice feedback questions. Using a computer randomization program, participants were randomly allocated to either the Compassionate Mind Training group (CMT group) or the Wait-List Control group (WLC group) on a 1:1 ratio. The CMT group completed the measures, in the same order, immediately after engaging in the 2-week CMT (T2), and again at a 2-week follow-up (T3). The WLC group completed the measures, in the same order, after a 2-week waiting period (T2) and immediately after completing the 2-week CMT (T3) (Table 1).

**Table 1:** Timeline across the two groups

Time 1 (T1)	Group		Time 2 (T2)		Time 3 (T3)
Baseline 1: Before CMT	CMT	Two-week CMT	Post-intervention: Immediately after CMT	-	Follow-up: Two weeks after CMT
Baseline 1: Before CMT	WLC	-	Baseline 2: Two weeks after baseline 1	Two-week CMT	Post-intervention: Immediately after CMT

This study used an English version of the CMT scripts developed for a 2-week CMT by Matos et al. (2017a) and translated to English and converted into audio recordings for UK use by Atuk (2020). This study also incorporated a psychoeducation video, converted from Matos and colleagues’ (2017a) psychoeducation booklet. The CMT scripts included the following practices:

1. Postures and Facial Expressions and Vocal Tones (PFEVT)
2. Mindfulness (M)
3. Soothing Rhythm Breathing (SRB)
4. Building and Cultivating Your Compassionate Self (BCYCS)
5. Compassion for a Close Person (CCP)
6. Compassion for the Self (CFTS)

Over 2 weeks, participants engaged in one video or audio material every day lasting no longer than 30 min (Table 2). Qualtrics online survey platform was used to deliver the CMT practices online. This software also generated a daily reminder for participants to practice the CMT tasks.

**Table 2:** Two-week CMT as informed by the study manual of Matos et al. (2017a)

Day 1 – Psychoeducation	Day 8 – CFTS
Day 2 – PFEVT and SRB	Day 9 – BCYCS
Day 3 – M and PFEVT	Day 10 – CCP
Day 4 – SRB and M	Day 11 – CFTS
Day 5 – Psychoeducation	Day 12 – BCYCS
Day 6 – BCYCS	Day 13 – CCP
Day 7 – CCP	Day 14 – CTFS

*PFEVT*, Postures and Facial Expressions and Vocal Tones; *SRB*, Soothing Rhythm Breathing; *M*, Mindfulness; *BCYCS*, Building and Cultivating Your Compassionate Self; *CCP*, Compassion for a Close Person; *CFTS*, Compassion for the Self

The practices comprised a psychoeducation session, which introduced participants to the concept of compassion. Other materials incorporated CMT practices that were aimed to facilitate a soothing rhythm breathing (Matos et al., 2017a), friendly facial expressions, and voice tones that would establish a compassionate atmosphere (Matos et al., 2017a; Porges, 2007). Additionally, there were practices aimed at increasing mindfulness and attention to one’s presence and mental state, and practices aimed at cultivating self-compassion and compassion to others via encouragement of wisdom, strength, and commitment (Matos et al., 2017a). CMT practices also aimed to increase participants’ reception towards compassion from others, by incorporating an imagery exercise, where participants were encouraged to develop a compassionate image of a caring other. Practices included exercises to help participants utilize compassion as a tool for dealing with distress and reducing self-criticism (Gilbert & Choden, 2013: Matos et al., 2017a).

### Measures

Participants completed a demographic form, in which they were required to identify their nationality (Sri Lankan vs. UK), religion (if any), age, and gender. Demographic factors were obtained to explore whether these were affecting the levels of compassion.

Next, they completed the Compassionate Engagement and Actions Scale (CEAS: Gilbert et al., 2017, 2017a; Gilbert et al., 2017b). This scale assesses compassion across the three flows of compassion: self-compassion (*α* = 0.91 and *ω* = 0.91: SL group, *α* = 0.89, *ω* = 0.90: UK group at baseline-1), compassion towards others (*α* = 0.93 and *ω* = 0.93: SL, *α* = 0.92 and *ω* = 0.92: UK), and compassion from others (*α* = 0.93 and *ω* = 0.93: SL, *α* = 0.94 and *ω* = 0.94: UK) and indicated excellent reliability at baseline-1. Each flow was measured using 13 items with answers ranging on a Likert scale from 1 (*never*) to 10 (*always*). This scale was used as it measures all three flows of compassion and to explore whether the three flows are correlated and would increase upon practicing CMT.

The Fears of Compassion Scales (FOCS: Gilbert et al., 2011) were used to measure fears across the three flows of compassion. Statements on this scale range on a 5-point Likert from 0 (*don’t agree at all*) to 4 (*completely agree*). This scale indicated a good reliability across Sri Lankan and UK samples (*α* = 0.78 and *ω* = 0.77: SL, *α* = 0.86 and *ω* = 0.86: UK for fear of self-compassion, *α* = 0.89 and *ω* = 0.89: SL, *α* = 0.92 and *ω* = 0.92: UK for fear of compassion from others, and *α* = 0.92 and *ω* = 0.92: SL, *α* = 0.95 and *ω* = 0.95: UK for fear of compassion towards others). This scale was used to explore whether the fear of compassion across the three flows inhibits participants’ compassion levels and whether these would decrease after practicing CMT.

The Forms of Self-Criticising/Attacking and Self-Reassuring Scale (FSCRS: Gilbert et al., 2004) was used to assess participants’ self-critical and self-reassuring responses to adverse experiences, measured on three dimensions—*inadequate-self*, *reassured-self*, and *hated-self*—using a 22-item Likert scale ranging from 1 (*not at all like me*) to 4 (*extremely like me*). A good reliability was indicated for all three dimensions of this scale at baseline-1 (*α* = 0.78 and *ω* = 0.78: SL and *α* = 0.90 and *ω* = 0.90: UK for inadequate-self, *α* = 0.82 and *ω* = 0.82: SL, *α* = 0.90 and *ω* = 0.90: UK for reassured self, and α = 0.82 and ω = 0.83: SL, α = 0.88 and ω = 0.88: UK for hated self). This scale was used to explore whether practicing CMT would increase self-reassurance and decrease self-criticism (as measured using inadequate-self and hated-self dimensions).

The Others as Shamer Scale (OAS: Allan et al., 1994) was used to understand participants’ perception of how others view them, also known as *external shame*. OAS scale has 18 items on a 5-point Likert scale that range from 0 (*never*) to 4 (*almost always*). A high reliability was indicated at baseline-1 (*α* = 0.94 and *ω* = 0.94: SL, *α* = 0.95 and *ω* = 0.95: UK). This scale was used to explore whether external shame participants perceive from their society would have an impact on their compassion levels and whether practicing CMT would decrease external shame (Goss et al., 1994).

Social Safeness and Pleasure Scale (SSPS: Gilbert et al., 2009) assessed how safe and warm people perceive their society to be. This scale consists of 11 items on a Likert scale ranging from 0 (*never*) to 4 (*almost all the time*), and acquired a good reliability (*α* = 0.89 and *ω* = 0.89: SL, *α* = 0.94 and *ω* = 0.94: UK). This scale was used to explore whether CMT would increase participants’ perceptions of social safeness and pleasure.

The Generalised Anxiety Disorder-7 Scale (GAD-7: Spitzer et al., 2006) and the Patient Health Questionnaire (PHQ-9: Kroenke et al., 2001) were used to measure participants’ anxiety and depression levels respectively. Both scales ranged on a Likert scale from 0 (*not at all*) to 3 (*nearly every day*) and indicated good internal reliability values (GAD-7: *α* = 0.83 and *ω* = 0.83 in SL, and *α* = 0.90 and *ω* = 0.90 in UK, PHQ: *α* = 0.87 and *ω* = 0.87 in SL, *α* = 0.90 and *ω* = 0.90 in UK). These scales were used to explore whether CMT would result in decreased psychopathology.

The Warwick-Edinburgh Mental Well-Being Scale (WEMWBS: Tennant et al., 2007) is a 14-item scale that assesses cognitive processes, feelings, and the quality of interpersonal relationships to measure well-being. This scale is measured on a 5-point Likert scale ranging from 1 (*none of the time*) to 5 (*all of the time*) and indicated a good internal reliability (*α* = 0.93 and *ω* = 0.93: SL, *α* = 0.93 and *ω* = 0.93: UK). This scale was used to explore whether CMT would increase participants’ well-being.

At the end of each day of the 2-week CMT, participants were requested to answer a question on how well they were able to engage in the CMT. The answers varied on a 5-point Likert scale from 1 (*not very well*) to 5 (*very well*). In addition, after completing the 2-week CMT, participants completed a feedback questionnaire regarding the CMT accessibility and feasibility, which contained 11 statements with answers ranging from 1 (*strongly disagree*) to 7 (*strongly agree*). Participants in the CMT group were sent an additional set of questions at follow-up, with four further statements regarding their experience of the CMT practices, with answers ranging from 1 (*strongly disagree*) to 7 *(strongly agree)*.

### Data Analyses

Data analyses were conducted using SPSS version 28. Prior to the analyses, data were checked for normality and any outliers by visually inspecting histograms, scatterplots, and boxplots. No extreme outliers (data points located outside the whiskers of the box plots) were found although the fears of compassion variable slightly deviated from normality due to a moderate positive skew and hated-self variable largely deviated from normality showing a multi-modal distribution. Skewness and Kurtosis values ranged between − 0.916 and 0.431 apart from fear of compassion from others and fear of self-compassion at Timepoint 3 which were − 1.067 and − 1.287 respectively. Data relating to fears of compassion variables were bootstrapped, and data relating to the hated-self outcome were re-coded into three categories. Chi-square and independent samples *t*-tests were performed to check for any differences between the two countries (Sri Lanka and UK) and the two conditions (CMT and WLC) at baseline-1 (T1).

To test the efficacy of the CMT on the two groups across time, a 2 × 2 mixed ANOVA design was employed with the two conditions (CMT vs WLC) as the between-group factor, and time (T1 and T2) as the within-group factor. Where significant time × group interactions were found, pairwise comparisons were further explored to identify which group may have significantly improved post CMT. The analyses were conducted using both intention to treat (ITT) and per-protocol (PP) analyses (for both countries separately) to look for effects based on randomization (ITT) as well as by adherence (PP). Post-CMT efficacy of the WLC group (T3) was not analyzed as there was not enough data to conduct a meaningful analysis (*n* = 0 in the Sri Lankan and *n* = 17 in the UK WLC groups at T3).

Next, analyses were conducted to investigate whether the efficacy of CMT was maintained at follow-up 2 weeks after completing the CMT (at T3). As only participants in the CMT group were required to complete this stage, a repeated-measures ANOVA was carried out on the CMT group in relation to the three time points (T1: before CMT, T2: immediately post CMT, and T3; 2 weeks post CMT). The Greenhouse–Geisser correction was used for *F*-test comparisons when sphericity was not met. Only per-protocol analyses were conducted, to see if there were any changes at follow-up in the intervention group.

## Results

### Differences Between Countries and Groups at Baseline (T1)

A majority of the participants were female in both countries (59.5% in the SL sample and 81.2% in the UK sample). There was a significant difference between age *X*^2^(4) = 219.95, *p* < 0.001, proportion of males and females in each sample *X*^2^(2) = 32.70, *p* < 0.001, and religion *X*^*2*^(7) = 270.23, *p* < 0.001, with a majority of the Sri Lankans being aged 25–34 years (55.6%) and self-identifying as Buddhist (51.7%), and a majority of the UK participants being aged 18–24 years (80.4%) and self-identifying as atheist (30.2%). See Table 3.

**Table 3:** Demographic information of Sri Lankan and UK participants

	Sri Lankan sample			UK sample		
	CMT group *n* = 119 (51.3%)	WLC group *n* = 113 (48.7%)	Total *n* = 232	CMT group *n* = 125 (51.0%)	WLC group *n* = 120 (49.0%)	Total *n* = 245
Gender						
Male	45 (37.8%)	49 (43.4%)	94 (40.5%)	22 (17.6%)	21 (17.5%)	43 (17.6%)
Female	74 (62.2%)	64 (56.6%)	138 (59.5%)	100 (80%)	99 (82.5%)	199 (81.2%)
Other	-	-		3 (2.4%)		3 (1.2%)
Age (years)						
18–24	23 (19.3%)	13 (11.5%)	36 (15.5%)	99 (79.2%)	98 (81.7%)	197 (80.4%)
25–34	65 (54.6%)	64 (56.6%)	129 (55.6%)	20 (16.0%)	14 (11.7%)	34 (13.9%)
35–44	30 (25.2%)	35 (31.0%)	65 (28%)	2 (1.6%)	4 (3.3%)	6 (2.4%)
45–54	1 (0.8%)		1 (0.4%)	4 (3.2%)	3 (2.5%)	7 (2.9%)
55–64	-	1 (0.9%)	1 (0.4%)	-	1 (0.8%)	1 (0.4%)
Religion						
Agnostic	-	-	-	21 (16.8%)	17 (14.2%)	38 (15.5%)
Atheist	-	-	-	34 (27.2%)	40 (33.3%)	74 (30.2%)
Buddhist	65 (54.6%)	55 (48.7%)	120 (51.7%)	2 (1.6%)	-	2 (0.8%)
Catholic	12 (10.1%)	12 (10.6%)	24 (10.3%)	18 (14.4%)	21 (17.5%)	39 (15.9%)
Christian	15 (12.6%)	16 (14.2%)	31 (13.4%)	20 (16.0%)	25 (20.8%)	45 (18.4%)
Hindu	14 (11.8%)	24 (21.2%)	38 (16.4%)	7 (5.6%)	5 (4.2%)	12 (4.9%)
Muslim	13 (10.9%)	6 (5.3%)	19 (8.2%)	5 (4%)	7 (5.8%)	12 (4.9%)
Other	-	-	-	18 (14.4%)	5 (4.2%)	23 (9.4%)

The *t*-tests at T1 showed significant differences in compassion to and from others, inadequate-self, and anxiety, which were all higher in UK participants. Fear of compassion to self/others and from others, reassured-self, hated-self, and external shame were all significantly higher in Sri Lankans. In the Sri Lankan sample, significant differences were indicated in fear of compassion from others and fear of self-compassion between the CMT and WLC groups, with the CMT group indicating greater scores. No significant differences were indicated at T1 between the CMT and WLC groups in the UK sample (Table 4).

**Table 4:** Sample characteristics at baseline-1

	Differences between countries			Differences between conditions					
	SL	UK	Difference tests	Sri Lankan sample			UK sample		
	*n* = 232	*n* = 245		CMT	WLC	Difference tests	CMT	WLC	Difference tests
	*M (SD)*	*M (SD)*		*M (SD)*	*M (SD)*		*M (SD)*	*M (SD)*	
Self-compassion	57.25 (16.79)	57.20 (13.57)	*t*(444) = 0.04, *p* = 0.972	58.79 (16.95)	55.63 (16.55)	*t*(230) = 1.44, *p* = 0.152	57.48 (13.01)	56.91 (14.10)	*t*(243) = 0.33, *p* = 0.742
Compassion to others	58.27 (16.73)	72.62 (15.14)	*t*(475) = − 9.83, ***p* < 0.001**	59.58 (16.48)	56.89 (16.96)	*t*(230) = 1.22, *p* = 0.222	71.98 (15.29)	73.28 (15.01)	*t*(243) = -0.67, *p* = 0.503
Compassion from others	55.74 (16.39)	59.08 (15.54)	*t*(475) = − 2.29, ***p* = 0.023**	56.51 (16.45)	54.92 (16.35)	*t*(230) = 0.74, *p* = 0.461	57.55 (15.49)	60.68 (15.50)	*t*(243) = -1.58, *p* = 0.116
Fear of compassion to others	31.12 (5.67)	28.02 (6.94)	*t*(456) = 5.36, ***p* < 0.001**	31.45 (5.67)	30.78 (5.67)	*t*(230) = 0.90, *p* = 0.372	28.16 (7.27)	27.88 (6.60)	*t*(243) = 0.321, *p* = 0.749
Fear of compassion from others	38.59 (8.78)	32.60 (10.18)	*t*(471) = 6.89, ***p* < 0.001**	39.87 (9.42)	37.24 (7.87)	*t*(230) = 2.30, ***p* = 0.022**	33.18 (10.57)	31.99 (9.76)	*t*(243) = 0.91, *p* = 0.364
Fear of self-compassion	42.11 (10.90)	36.76 (13.54)	*t*(463) = 4.76, ***p* < 0.001**	43.53 (10.89)	40.61 (10.75)	*t*(230) = 2.05, ***p* = 0.041**	36.89 (13.51)	36.63 (13.63)	*t*(243) = 0.15, *p* = 0.883
Inadequate self	17.44 (5.31)	21.36 (7.36)	*t*(444) = − 6.68, ***p* < 0.001**	17.93 (5.38)	16.93 (5.22)	*t*(230) = 1.44, *p* = 0.151	21.51 (7.71)	21.19 (7.02)	*t*(243) = 0.34, *p* = 0.734
Reassured self	17.80 (5.12)	16.19 (5.89)	*t*(475) = 3.18, ***p* = 0.002**	18.26 (4.92)	17.32 (5.30)	*t*(230) = 1.41, *p* = 0.162	16.45 (5.97)	15.93 (5.81)	*t*(243) = 0.70, *p* = 0.488
Hated self	2.04 (.66)	1.91 (0.75)	*t*(473) = 2.12, ***p* = 0.035**	2.12 (0.70)	1.96 (0.61)	*t*(228) = 1.77, *p* = 0.078	1.94 (0.75)	1.88 (0.75)	*t*(243) = 0.64, *p* = 0.525
External shame	49.81 (12.16)	46.19 (13.29)	*t*(475) = 3.10, ***p* = 0.002**	50.85 (12.56)	48.71 (11.67)	*t*(230) = 1.34, *p* = 0.181	46.36 (13.57)	46.01 (13.05)	*t*(243) = 0.21, *p* = 0.836
Social safeness	34.49 (7.07)	35.81 (8.13)	*t*(472) = − 1.90, *p* = 0.058	35.09 (7.19)	33.85 (6.92)	*t*(230) = 1.34, *p* = 0.181	35.95 (8.18)	35.66 (8.11)	*t*(243) = 0.28, *p* = 0.778
Anxiety	15.93 (3.75)	17.02 (5.32)	*t*(439) = − 2.61, ***p* = 0.010**	16.00 (4.00)	15.85 (3.48)	*t*(230) = 0.31, *p* = 0.761	17.10 (5.28)	16.94 (5.39)	*t*(243) = 0.23, *p* = 0.821
Depression	19.45 (5.04)	20.23 (6.18)	*t*(465) = − 1.52, *p* = 0.128	19.54 (5.29)	19.35 (4.78)	*t*(230) = 0.28, *p* = 0.782	19.94 (6.05)	20.54 (6.32)	*t*(243) =—0.77, *p* = 0.444
Well-being	43.72 (9.22)	42.08 (9.18)	*t*(475) = 1.94, *p* = 0.052	43.97 (9.87)	43.46 (8.52)	*t*(230) = 0.42, *p* = 0.677	42.56 (9.23)	41.58 (9.24)	*t*(243) = 0.83, *p* = 0.406

*SL*, Sri Lanka; *UK*, United Kingdom; *N*, number of participants; *M*, mean; *SD*, standard deviation; *CMT*, Compassionate Mind Training group; *WLC*, Wait-List Control group; bold numbers, significant values; non-bold values, non-significant values

### Efficacy of the CMT: Sri Lankan Sample

The results of the mixed factorial 2 × 2 ANOVA showed a significant time × group (T1 vs. T2; CMT vs. WLC) interaction among all three flows of compassion (self-compassion, compassion to others, and compassion from others), with small effect sizes in the ITT analysis and large effect sizes in the PP analysis.

In addition, significant interactions were found for fear of compassion from others, fear of self-compassion, inadequate-self, reassured-self, social safeness and pleasure, and well-being with small effects in the ITT and large effects in the PP in all interactions. This indicates that facilitators of compassion (reassured-self, social safeness and pleasure, and well-being) significantly increased and the inhibitors of compassion (fear of compassion from others, fear of self-compassion, and inadequate-self) were significantly reduced, when compared to the WLC group at T2 (see Table 5 for mean differences and ANOVA results of the ITT, and Table 6 for equivalent PP results).

**Table 5:** Pre-post intention to treat analyses of the Sri Lankan sample

Measure	Time	CMT group (T1 *n* = 119) *M (SD)*	WLC group (T1 *n* = 113) *M (SD)*	Tests of within-subject effects		Tests of between-subject effects
				Time	Time × group	Group
Self-Compassion	T1	58.79 (16.95)	55.63 (16.55)	*F*_(1, 230)_ = 6.41, ***p* = 0.012**, *η*_p_^2^ = 0.03	*F*_(1, 230)_ = 6.95, ***p* = 0.009**, *η*_p_^2^ = 0.03	*F*_(1, 230)_ = 4.52, ***p* = 0.035**, *η*_p_^2^ = 0.02
	T2	61.43 (16.48)	55.58 (16.39)			
Compassion to others	T1	59.58 (16.48)	56.89 (16.96)	*F*_(1, 230)_ = 8.30, ***p* = 0.004**, *η*_p_^2^ = 0.04	*F*_(1, 230)_ = 9.80, ***p* = 0.002**, *η*_p_^2^ = 0.04	*F*_(1, 230)_ = 4.08, ***p* = 0.045**, *η*_p_^2^ = 0.02
	T2	62.57 (15.63)	56.77 (16.68)			
Compassion from others	T1	56.52 (16.45)	54.92 (16.35)	*F*_(1, 230)_ = 5.72, ***p* = 0.018**, *η*_p_^2^ = 0.02	*F*_(1, 230)_ = 7.56, ***p* = 0.006**, *η*_p_^2^ = 0.03	*F*_(1, 230)_ = 2.17, *p* = 0.142, *η*_p_^2^ = 0.01
	T2	59.18 (15.45)	54.73 (16.19)			
Fear of compassion to others	T1	31.45 (5.67)	30.78 (5.67)	*F*_(1, 230)_ = 0.05, *p* = 0.827, *η*_p_^2^ = 0.00	*F*_(1, 230)_ = 0.26, *p* = 0.614, *η*_p_^2^ = 0.00	*F*_(1, 230)_ = 0.66, *p* = 0.416, *η*_p_^2^ = 0.00
	T2	31.40 (5.60)	30.88 (5.65)			
Fear of compassion from others	T1	39.87 (9.42)	37.24 (7.87)	*F*_(1, 230)_ = 0.96, *p* = 0.327, *η*_p_^2^ = 0.00	*F*_(1, 230)_ = 5.65, ***p* = 0.018**, *η*_p_^2^ = 0.02	*F*_(1, 230)_ = 3.79, *p* = 0.055, *η*_p_^2^ = 0.02
	T2	39.27 (9.43)	37.49 (7.96)			
Fear of self-compassion	T1	43.53 (10.89)	40.61 (10.75)	*F*_(1, 230)_ = 3.27, *p* = 0.072, *η*_p_^2^ = 0.01	*F*_(1, 230)_ = 9.47, ***p* = 0.002**, *η*_p_^2^ = 0.04	*F*_(1, 230)_ = 2.31, *p* = 0.130, *η*_p_^2^ = 0.01
	T2	42.24 (10.72)	40.95 (10.55)			
Inadequate self	T1	17.93 (5.38)	16.93 (5.22)	*F*_(1, 230)_ = 1.95, *p* = 0.164, *η*_p_^2^ = 0.01	*F*_(1, 230)_ = 5.98, ***p* = 0.015**, *η*_p_^2^ = 0.03	*F*_(1, 230)_ = 1.10, *p* = 0.297, *η*_p_^2^ = 0.01
	T2	17.48 (5.32)	17.05 (5.18)			
Reassured self	T1	18.26 (4.92)	17.32 (5.30)	*F*_(1, 230)_ = 4.84, ***p* = 0.029**, *η*_p_^2^ = 0.02	*F*_(1, 230)_ = 4.10, ***p* = 0.044**, *η*_p_^2^ = 0.02	*F*_(1, 230)_ = 3.65, *p* = 0.057, *η*_p_^2^ = 0.02
	T2	18.90 (4.90)	17.35 (5.30)			
Hated self	T1	2.12 (.70)	1.96 (.61)	*F*_(1, 230)_ = 6.11, ***p* = 0.014**, *η*_p_^2^ = 0.03	*F*_(1, 230)_ = 0.00, *p* = 0.949, *η*_p_^2^ = 0.00	*F*_(1, 230)_ = 3.22, *p* = 0.074, *η*_p_^2^ = 0.01
	T2	2.09 (0.70)	1.94 (0.60)			
External shame	T1	50.85 (12.56)	48.71 (11.67)	*F*_(1, 230)_ = 2.04, *p* = 0.155, *η*_*p*_^2^ = 0.01	*F*_(1, 230)_ = 0.02, p = 0.877, *η*_p_^2^ = 0.00	*F*_(1, 230)_ = 1.85, p = 0.175, *η*_p_^2^ = 0.01
	T2	50.76 (12.38)	48.59 (11.53)			
Social safeness	T1	35.09 (7.19)	33.85 (6.92)	*F*_(1, 230)_ = 6.13, ***p* = 0.014**, *η*_p_^2^ = 0.03	*F*_(1, 230)_ = 9.41, ***p* = 0.002**, *η*_p_^2^ = 0.04	*F*_(1, 230)_ = 4.70, ***p* = 0.031**, *η*_p_^2^ = 0.02
	T2	36.34 (7.15)	33.72 (6.69)			
Anxiety	T1	16.00 (4.00)	15.85 (3.48)	*F*_(1, 230)_ = 5.02, ***p* = 0.026**, *η*_p_^2^ = 0.02	*F*_(1, 230)_ = 1.91 *p* = 0.168, *η*_p_^2^ = 0.01	*F*_(1, 230)_ = 0.00, *p* = 0.963, *η*_p_^2^ = 0.00
	T2	15.66 (4.04)	15.77 (3.29)			
Depression	T1	19.54 (5.29)	19.35 (4.78)	*F*_(1, 230)_ = 0.09, *p* = 0.762, *η*_p_^2^ = 0.00	*F*_(1, 230)_ = 1.61, *p* = 0.206, *η*_p_^2^ = 0.01	*F*_(1, 230)_ = 0.02, *p* = 0.877, *η*_p_^2^ = 0.00
	T2	19.44 (5.30)	19.42 (4.82)			
Well-being	T1	43.97 (9.87)	43.46 (8.52)	*F*_(1, 230)_ = 3.78, *p* = 0.053, *η*_p_^2^ = 0.02	*F*_(1, 230)_ = 9.81 ***p* = 0.002**, *η*_p_^2^ = 0.04	*F*_(1, 230)_ = 1.28, *p* = 0.260, *η*_p_^2^ = 0.01
	T2	45.33 (9.95)	43.14 (8.71)			

*n*, number of participants; *M*, mean; *SD*, standard deviation; *T1*, Timepoint 1; *T2*, Timepoint 2; bold numbers, values that are significant; non-bold values, values that are non-significant

**Table 6:** Pre-post per protocol analyses of the Sri Lankan sample

Measure	Time	CMT group (T2n = 21) *M (SD)*	WLC group (T2n = 17) *M (SD)*	Tests of within-subject effects		Tests of between-subject effects
				Time	Time × group	Group
Self-compassion	T1	61.00 (21.06)	66.12 (13.20)	*F*_(1, 36)_ = 7.19, ***p* = 0.011**, *η*_p_^2^ = 0.17	*F*_(1, 36)_ = 7.90, ***p* = 0.008**, *η*_p_^2^ = 0.18	*F*_(1, 36)_ = 0.41, *p* = 0.529, *η*_p_^2^ = 0.01
	T2	75.95 (9.36)	65.76 (12.10)			
Compassion to others	T1	58.71 (21.95)	71.65 (12.20)	*F*_(1, 36)_ = 10.70, ***p* = 0.002**, *η*_p_^2^ = 0.23	*F*_(1, 36)_ = 13.00, ***p* < 0.001,** *η*_p_^2^ = 0.27	*F*_(1, 36)_ = 0.94, *p* = 0.338, *η*_p_^2^ = 0.03
	T2	75.67 (10.20)	70.82 (10.43)			
Compassion from others	T1	57.43 (22.38)	66.71 (9.40)	*F*_(1, 36)_ = 6.28, ***p* = 0.017**, *η*_p_^2^ = 0.15	*F*_(1, 36)_ = 8.71, ***p* = 0.006**, *η*_p_^2^ = 0.20	*F*_(1, 36)_ = 0.09, *p* = 0.770, *η*_p_^2^ = 0.00
	T2	72.57 (9.23)	65.47 (9.05)			
Fear of compassion to others	T1	33.05 (6.23)	33.65 (6.27)	*F*_(1, 36) =_ 0.07, *p* = 0.800, *η*_p_^2^ = 0.00	*F*_(1, 36)_ = 0.27, *p* = 0.610, *η*_p_^2^ = 0.01	*F*_(1, 36)_ = 0.38, *p* = 543, *η*_p_^2^ = 0.01
	T2	32.81 (5.88)	34.35 (5.78)			
Fear of compassion from others	T1	44.10 (9.19)	37.47 (8.17)	*F*_(1, 36)_ = 0.70, *p* = 0.407, *η*_p_^2^ = 0.02	*F*_(1, 36)_ = 5.91, ***p* = 0.020**, *η*_p_^2^ = 0.14	*F*_(1, 36)_ = 2.16, *p* = 0.151, *η*_p_^2^ = 0.06
	T2	40.71 (10.27)	39.12 (8.52)			
Fear of self-compassion	T1	48.14 (12.67)	39.47 (13.09)	*F*_(1, 36)_ = 3.21, *p* = 0.082, *η*_p_^2^ = 0.08	*F*_(1, 36)_ = 11.29, ***p* = 0.002**, *η*_p_^2^ = 0.24	*F*_(1, 36)_ = 1.01, *p* = 0.322, *η*_p_^2^ = 0.03
	T2	40.81 (12.77)	41.71 (11.94)			
Inadequate self	T1	21.05 (5.00)	20.76 (5.40)	*F*_(1, 36)_ = 1.67, *p* = 0.205, *η*_p_^2^ = 0.04	*F*_(1, 36)_ = 6.29, ***p* = 0.017**, *η*_p_^2^ = 0.15	*F*_(1, 36)_ = 0.84, *p* = 0.365, *η*_p_^2^ = 0.02
	T2	18.48 (5.69)	21.59 (4.37)			
Reassured self	T1	18.41 (6.25)	20.81 (5.24)	*F*_(1, 41)_ = 5.68, ***p* = 0.022,** *η*_p_^2^ = 0.12	*F*_(1, 41)_ = 4.81**, *p* = 0.034**, *η*_p_^2^ = 0.11	*F*_(1, 41)_ = 0.25, *p* = 0.621, *η*_p_^2^ = 0.01
	T2	21.86 (5.20)	20.95 (5.15)			
Hated self	T1	2.33 (.80)	1.89 (.74)	*F*_(1, 38)_ = 6.73, ***p* = 0.013**, *η*_p_^2^ = 0.15	*F*_(1, 38)_ = 0.02, *p* = 0.898, *η*_p_^2^ = 0.00	*F*_(1, 38)_ = 3.70, *p* = 0.062, *η*_p_^2^ = 0.09
	T2	2.19 (.81)	1.74 (.65)			
External shame	T1	54.09 (13.18)	48.29 (13.88)	*F*_(1, 37)_ = 2.11, *p* = 0.154, *η*_p_^2^ = 0.05	*F*_(1, 37)_ = 0.09, *p* = 0.763, *η*_p_^2^ = 0.00	*F*_(1, 37)_ = 2.00, *p* = 0.166, *η*_p_^2^ = 0.05
	T2	53.59 (12.33)	47.53 (12.95)			
Social safeness	T1	34.62 (7.90)	38.71 (6.51)	*F*_(1, 36)_ = 7.08, ***p* = 0.012**, *η*_p_^2^ = 0.16	*F*_(1, 36)_ = 11.72, ***p* = 0.002**, *η*_p_^2^ = 0.25	*F*_(1, 36)_ = 0.01, *p* = 0.943, *η*_p_^2^ = 0.00
	T2	41.67 (4.87)	37.82 (5.34)			
Anxiety	T1	18.05 (3.92)	16.12 (4.74)	*F*_(1, 36)_ = 5.11, ***p* = 0.030,** *η*_p_^2^ = 0.12	*F*_(1, 36)_ = 1.63, *p* = 0.210, *η*_p_^2^ = 0.04	*F*_(1, 36)_ = 0.93, *p* = 0.342, *η*_p_^2^ = 0.03
	T2	16.14 (4.72)	15.59 (3.64)			
Depression	T1	21.14 (6.42)	19.47 (7.02)	*F*_(1, 36)_ = 0.046, *p* = 0.841, *η*_p_^2^ = 0.00	*F*_(1, 23)_ = 1.55, *p* = 0.222, *η*_p_^2^ = 0.04	*F*_(1, 36)_ = 0.29, *p* = 0.591, *η*_p_^2^ = 0.01
	T2	20.57 (6.61)	19.88 (7.18)			
Well-being	T1	45.62 (10.45)	47.12 (8.35)	*F*_(1, 36)_ = 3.86, *p* = 0.057, *η*_p_^2^ = 0.10	*F*_(1, 36)_ = 11.92, ***p* = 0.001**, *η*_p_^2^ = 0.25	*F*_(1, 36)_ = 1.79, *p* = 0.189, *η*_p_^2^ = 0.05
	T2	53.33 (6.32)	45.00 (10.24)			

*n*, number of participants; *M*, mean; *SD*, standard deviation; *T1*, Timepoint 1; *T2*, Timepoint 2; bold numbers, values that are significant; non-bold values, values that are non-significant

### Efficacy of the CMT: UK Sample

The results of the mixed factorial 2 × 2 ANOVA showed a significant time × group (T1 vs. T2; CMT vs. WLC) interaction among all three flows of compassion with a large effect size for self-compassion, and small effect sizes for compassion to others and compassion from others in the ITT and PP analyses.

Significant interactions were reported for all three types of fears of compassion (fear of compassion to others with a small effect size in ITT and medium effect size in PP, fear of compassion from others with small effect sizes in ITT and PP, and fear of self-compassion with small effect sizes in ITT and PP). Significant interactions with small effect sizes were also reported for external shame, anxiety, and depression in the ITT and PP analyses, and a significant interaction with a medium effect size was found for inadequate self in the ITT and PP analyses. See Table 7 for ITT and Table 8 for PP analyses.

**Table 7:** Pre-post intention to treat analyses of the UK sample

Measure	Time	CMT group (T1n = 125) *M (SD)*	WLC Group (T1n = 120) *M (SD)*	Tests of within-subject effects		Tests of between-subject effects
				Time	Time × group	Group
Self-compassion	T1	57.48 (13.08)	56.91 (14.10)	*F*_(1, 243)_ = 14.51, ***p* < 0.001**, *η*_p_^2^ = 0.06	*F*_(1, 243)_ = 34.22, ***p* < 0.001**, *η*_p_^2^ = 0.12	*F*_(1, 243)_ = 4.37, ***p* = 0.038**, *η*_p_^2^ = 0.02
	T2	62.49 (14.13)	55.85 (15.04)			
Compassion to others	T1	71. 98 (15.29)	73.28 (15.01)	*F*_(1, 243)_ = 7.57, ***p* = 0.006**, *η*_p_^2^ = 0.03	*F*_(1, 243)_ = 7.09, ***p* = 0.008**, *η*_p_^2^ = 0.03	*F*_(1, 243)_ = 0.02, *p* = 0.880, *η*_p_^2^ = 0.00
	TT2	74.04 (15.35)	73.32 (14.90)			
Compassion from others	TT1	57.55 (15.49)	60.68 (15.50)	*F*_(1, 243)_ = 9.23, ***p* = 0.003**, *η*_p_^2^ = 0.04	*F*_(1, 243)_ = 7.67, ***p* = 0.006**, *η*_p_^2^ = 0.03	*F*_(1, 243)_ = 0.61, *p* = 0.436, *η*_p_^2^ = 0.00
	TT2	60.97 (16.00)	60.83 (16.70)			
Fear of compassion to others	TT1	28.16 (7.27)	27.87 (6.60)	*F*_(1, 243)_ = 13.79, ***p* < 0.001**, *η*_p_^2^ = 0.05	*F*_(1, 243)_ = 10.15, ***p* = 0.002**, *η*_p_^2^ = 0.04	*F*_(1, 243)_ = 0.49, *p* = 0.484, *η*_p_^2^ = 0.00
	TT2	26.20 (8.16)	27.73 (6.90)			
Fear of compassion from others	T TT1	0.57)	31.99 (9.76)	*F*_(1, 243)_ = 11.54, ***p* < 0.001**, *η*_p_^2^ = 0.05	*F*_(1, 243)_ = 6.71, ***p* = 0.010**, *η*_p_^2^ = 0.03	*F*_(1, 243)_ = 0.09, *p* = 0.765, *η*_p_^2^ = 0.00
	TT2	31.32 (10.87)	31.74 (9.70)			
Fear of self-compassion	TT1	36.89 (13.51)	36.63 (13.63)	*F*_(1, 243)_ = 8.72, ***p* = 0.003**, *η*_p_^2^ = 0.04	*F*_(1, 243)_ = 8.60, ***p* = 0.004**, *η*_p_^2^ = 0.03	*F*_(1, 243)_ = 0.34, *p* = 0.563, *η*_p_^2^ = 0.00
	TT2	34.38 (14.61)	36.63 (13.67)			
Inadequate self	TT1	21.51 (7.71)	21.34 (6.96)	*F*_(1, 243)_ = 22.75, ***p* < 0.001**, *η*_p_^2^ = 0.09	*F*_(1, 243)_ = 15.08, ***p* < 0.001**, *η*_p_^2^ = 0.06	*F*_(1, 243)_ = 1.14, *p* = 0.287, *η*_p_^2^ = 0.00
	TT2	18.99 (7.86)	21.06 (7.03)			
Reassure self	TT1	16.45 (5.97)	15.93 (5.81)	*F*_(1, 243)_ = 3.83, *p* = 0.051, *η*_p_^2^ = 0.02	*F*_(1, 243)_ = 0.01, *p* = 0.919, *η*_p_^2^ = 0.00	*F*_(1, 243)_ = 0.55, *p* = 0.459, *η*_p_^2^ = 0.00
	TT2	16.74 (5.58)	16.19 (5.79)			
Hated self	TT1	1.94 (0.75)	1.88 (0.75)	*F*_(1, 243)_ = 0.99, *p* = 0.321, *η*_p_^2^ = 0.00	*F*_(1, 243)_ = 0.03, *p* = 0.858, *η*_p_^2^ = 0.00	*F*_(1, 243)_ = 0.47, *p* = 0.495, *η*_p_^2^ = 0.00
	TT2	1.96 (0.78)	1.89 (0.75)			
External shame	TT1	46.36 (13.57)	46.01 (13.05)	*F*_(1, 243)_ = 8.49, ***p* = 0.004**, *η*_p_^2^ = 0.03	*F*_(1, 243)_ = 5.81, ***p* = 0.017**, *η*_p_^2^ = 0.02	*F*_(1, 243)_ = 0.17, *p* = 0.682, *η*_p_^2^ = 0.00
	TT2	44.07 (14.46)	45.79 (12.76)			
Social safeness	TT1	35.95 (8.18)	35.66 (8.11)	*F*_(1, 243)_ = 19.52, ***p* < 0.001**, *η*_p_^2^ = 0.07	*F*_(1, 243)_ = 2.61, *p* = 0.107, *η*_p_^2^ = 0.01	*F*_(1, 243)_ = 0.57, *p* = 0.451, *η*_p_^2^ = 0.00
	TT2	37.73 (8.54)	36.48 (8.36)			
Anxiety	TT1	17.10 (5.28)	16.94 (5.39)	*F*_(1, 243)_ = 16.84, ***p* < 0.001**, *η*_p_^2^ = 0.07	*F*_(1, 243)_ = 8.45, ***p* = 0.004**, *η*_p_^2^ = 0.03	*F*_(1, 243)_ = 0.55, *p* = 0.459, *η*_p_^2^ = 0.00
	TT2	15.58 (5.17)	16.68 (5.21)			
Depression	TT1	19.94 (6.05)	20.54 (6.32)	*F*_(1, 243)_ = 15.72, ***p* < 0.001**, *η*_p_^2^ = 0.06	*F*_(1, 243)_ = 6.39, ***p* = 0.012**, *η*_p_^2^ = 0.03	*F*_(1, 243)_ = 2.37, *p* = 0.125, *η*_p_^2^ = 0.01
	TT2	18.50 (5.81)	20.22 (6.47)			
Well-being	TT1	42.56 (9.13)	41.58 (9.24)	*F*_(1, 243)_ = 3.50 *p* = 0.062, *η*_p_^2^ = 0.01	*F*_(1, 243)_ = 1.60, *p* = 0.207, *η*_p_^2^ = 0.01	*F*_(1, 243)_ = 1.47, *p* = 0.227, *η*_p_^2^ = 0.01
	TT2	43.64 (10.11)	41.79 (9.53)			

*n*, number of participants; *M*, mean; *SD*, standard deviation; *T1*, Timepoint 1; *T2*, Timepoint 2; bold numbers, values that are significant; non-bold values, values that are non-significant

**Table 8:** Pre-post per protocol analyses of the UK sample

Measure	Time	CMT group (T2n = 73) *M (SD)*	WLC group (T2n = 54) *M (SD)*	Tests of within-subject effects		Tests of between-subject effects
				Time	Time × group	Group
Self-compassion	T1	57.99 (12.76)	58.24 (13.54)	*F*_(1, 125)_ = 11.05, ***p* = 0.001**, *η*_p_^2^ = 0.08	*F*_(1, 125)_ = 34.06, ***p* < 0.001**, *η*_p_^2^ = 0.21	*F*_(1, 125)_ = 5.25, ***p* = 0.024**, *η*_p_^2^ = 0.04
	T2	66.56 (13.12)	65.89 (15.74)			
Compassion to others	T1	74.82 (12.12)	75.28 (11.53)	*F*_(1, 128)_ = 6.44, ***p* = 0.012**, *η*_p_^2^ = 0.05	*F*_(1, 128)_ = 5.94, ***p* = 0.016**, *η*_p_^2^ = 0.04	*F*_(1, 128)_ = 0.44, *p* = 0.509, *η*_p_^2^ = 0.00
	T2	78.34 (11.15)	75.35 (11.22)			
Compassion from others	T1	58.32 (15.01)	61.53 (16.16)	*F*_(1, 128)_ = 8.01, ***p* = 0.005**, *η*_p_^2^ = 0.06	*F*_(1, 128)_ = 6.38, ***p* = 0.013**, *η*_p_^2^ = 0.05	*F*_(1, 128)_ = 0.03, *p* = 0.859, *η*_p_^2^ = 0.00
	T2	64.16 (15.16)	61.86 (16.53)			
Fear of compassion to others	T1	28.23 (7.21)	27.56 (6.74)	*F*_(1, 128)_ = 12.39, ***p* < 0.001**, *η*_p_^2^ = 0.09	*F*_(1, 128)_ = 8.50, ***p* = 0.004**, *η*_p_^2^ = 0.06	*F*_(1, 128)_ = 0.48, *p* = 0.488, *η*_p_^2^ = 0.00
	T2	24.88 (8.44)	27.25 (7.32)			
Fear of compassion from others	T1	31.34 (9.99)	31.21 (9.57)	*F*_(1, 128)_ = 10.40, ***p* = 0.002**, *η*_p_^2^ = 0.08	*F*_(1, 128)_ = 5.33, ***p* = 0.023**, *η*_p_^2^ = 0.04	*F*_(1, 128)_ = 0.55 *p* = 0.461, *η*_p_^2^ = 0.00
	T2	28.16 (9.73)	30.68 (9.39)			
Fear of self-compassion	T1	35.33 (13.15)	34.75 (13.79)	*F*_(1, 128)_ = 7.43, ***p* = 0.007**, *η*_p_^2^ = 0.06	*F*_(1, 128)_ = 7.31, ***p* = 0.008**, *η*_p_^2^ = 0.05	*F*_(1, 128)_ = 0.47, *p* = 0.496, *η*_p_^2^ = 0.00
	T2	31.03 (14.32)	34.74 (13.80)			
Inadequate self	T1	22.12 (8.15)	21.84 (6.79)	*F*_(1, 128)_ = 21.62, ***p* < 0.001**, *η*_p_^2^ = 0.15	*F*_(1, 128)_ = 13.02, ***p* < 0.001**, *η*_p_^2^ = 0.09	*F*_(1, 128)_ = 1.66, *p* = 0.200, *η*_p_^2^ = 0.01
	T2	17.81 (8.24)	21.30 (6.96)			
Reassure self	T1	16.36 (6.47)	15.77 (6.05)	*F*_(1, 128)_ = 3.86, *p* = 0.052**,** *η*_p_^2^ = 0.03	*F*_(1, 128)_ = 0.01, *p* = 0.920, *η*_p_^2^ = 0.00	*F*_(1, 128)_ = 0.29, *p* = 0.594 *η*_p_^2^ = 0.00
	T2	16.86 (5.83)	16.33 (6.01)			
Hated self	T1	1.90 (0.77)	1.70 (0.73)	*F*_(1, 128)_ = 0.96, *p* = 0.329, *η*_p_^2^ = 0.01	*F*_(1, 128)_ = 0.01, *p* = 0.939, *η*_p_^2^ = 0.00	*F*_(1, 128)_ = 2.49, *p* = 0.117, *η*_p_^2^ = 0.02
	T2	1.95 (.81)	1.74 (.74)			
External shame	T1	45.01 (13.42)	43.42 (12.29)	*F*_(1, 128)_ = 7.45, ***p* = 0.007**, *η*_p_^2^ = 0.06	*F*_(1, 128)_ = 4.67, ***p* = 0.033**, *η*_p_^2^ = 0.04	*F*_(1, 128)_ = 0.00, *p* = 0.949, *η*_p_^2^ = 0.00
	T2	41.10 (14.35)	42.96 (11.53)			
Social safeness	T1	35.89 (8.52)	35.51 (7.91)	*F*_(1, 128)_ = 19.55, ***p* < 0.001**, *η*_p_^2^ = 0.13	*F*_(1, 128)_ = 1.46, *p* = 0.230, *η*_p_^2^ = 0.01	*F*_(1, 128)_ = 0.55, *p* = 0.461, *η*_p_^2^ = 0.00
	T2	38.93 (8.92)	37.25 (8.38)			
Anxiety	T1	17.18 (5.37)	17.42 (5.57)	*F*_(1, 128)_ = 15.73, ***p* < 0.001,** *η*_p_^2^ = 0.11	*F*_(1, 128)_ = 6.71, ***p* = 0.011**, *η*_p_^2^ = 0.05	*F*_(1, 128)_ = 2.25, *p* = 0.136, *η*_p_^2^ = 0.02
	T2	14.59 (4.94)	16.88 (5.23)			
Depression	T1	19.85 (6.03)	20.72 (5.99)	*F*_(1, 128)_ = 14.77, ***p* < 0.001**, *η*_p_^2^ = 0.10	*F*_(1, 128)_ = 4.84, ***p* = 0.030**, *η*_p_^2^ = 0.04	*F*_(1, 128)_ = 3.37, *p* = 0.069, *η*_p_^2^ = 0.03
	T2	17.40 (5.34)	20.05 (6.33)			
Well-being	T1	43.26 (8.77)	41.53 (8.00)	*F*_(1, 128)_ = 3.09, *p* = 0.081, *η*_p_^2^ = 0.02	*F*_(1, 128)_ = 1.18*, p* = *0.*280, *η*_p_^2^ = 0.01	*F*_(1, 128)_ = 2.79, *p* = 0.097, *η*_p_^2^ = 0.02
	T2	45.11 (10.25)	41.96 (8.70)			

*n*, number of participants; *M*, mean; *SD*, standard deviation; *T1*, Timepoint 1; *T2*, Timepoint 2; bold numbers, values that are significant; non-bold values, values that are non-significant

### Maintenance of Efficacy of the CMT at Follow-up: Sri Lankan Sample

A repeated-measures ANOVA was used to investigate whether the efficacy of CMT was maintained at follow-up (T3) 2 weeks after completing the CMT. Results indicated that all changes observed at post-intervention were maintained at follow-up.

Results for each of the three flows of compassion showed self-compassion, compassion to others, and compassion from others differed significantly across the three time points, with large effect sizes. Bonferroni corrected tests indicated that all three flows of compassion increased significantly from T1 (baseline 1) to T2 (post-intervention), and T1 (baseline 1) to T3 (follow-up), but not from T2 (post-intervention) to T3 (follow-up). Similarly, fear of compassion from others, fear of self-compassion, and inadequate self also changed significantly with large effects across the three time points, again with significant changes between T1 to T2 and T1 to T3, but not from T2 to T3. Although no changes were reported post CMT, anxiety and depression scores indicated a significant change with a large effect across time at follow-up, with only a significant change from T1 to T3 in anxiety (although there was an overall significant ANOVA for depression, none of the pairwise comparisons was significant). Significant large effects were indicated for reassured-self with a significant change from T1 to T2 but not from T1 to T3 or T2 to T3. Social safeness and well-being outcomes changed significantly with large effects, indicating significant increases from T1 to T2, and T1 to T3, but not from T2 to T3 (Table 9).

**Table 9:** Changes across time in the Sri Lankan sample

Measure	Time	CMT group (*n*_T1_ = 119, *n*_T2_ = 21, *n*_T3_ = 19) M (SD)	Tests of within-subject effects	T1 vs T2		T1 vs T3		T2 vs T3	
			Time	*MD*	Sig	*MD*	Sig	*MD*	Sig
Self-compassion	T1	60.32 (22.04)	*F*_(1, 19)_ = 9.37, ***p* = 0.006**, *η*_p_^2^ = 0.34	− 15.42	**0.013**	− 14.74	**0.027**	0.68	1.000
	T2	75.74 (9.47)							
	T3	75.05 (10.15)							
Compassion to others	T1	57.26 (22.49)	*F*_(1, 19)_ = 14.37, ***p* = 0.001**, *η*_p_^2^ = 0.44	− 17.37	**0.003**	− 17.16	**0.005**	0.21	1.000
	T2	74.63 (10.17)							
	T3	74.42 (11.45)							
Compassion from others	T1	57.37 (23.59)	*F*_(1, 19)_ = 7.90, ***p* = 0.010**, *η*_p_^2^ = 0.31	− 15.26	**0.030**	− 15.05	**0.037**	0.21	1.000
	T2	72.63 (9.73)							
	T3	72.42 (10.80)							
Fear of compassion to others	T1	32.74 (6.47)	*F*_(1, 24)_ = 0.06, *p* = 0.879, *η*_p_^2^ = 0.00	− 0.21	1.0	0.16	1.0	0.37	1.000
	T2	32.95 (5.65)							
	T3	32.58 (7.17)							
Fear of compassion from others	T1	44.74 (9.30)	*F*_(1, 21)_ = 8.17, ***p* = 0.007**, η_p_^2^ = 0.31	3.84	**0.038**	4.68	**0.022**	0.84	0.346
	T2	40.89 (10.28)							
	T3	40.05 (11.56)							
Fear of self-compassion	T1	48.89 (12.77)	*F*_(1, 19)_ = 14.62, ***p* < 0.001**, *η*_p_^2^ = 0.45	7.74	**0.005**	8.63	**0.002**	0.90	0.189
	T2	41.16 (13.10)							
	T3	40.26 (13.28)							
Inadequate self	T1	21.11 (5.24)	*F*_(1, 26)_ = 9.29, ***p* = 0.002**, *η*_p_^2^ = 0.34	2.84	**0.010**	3.21	**0.015**	0.37	1.000
	T2	18.26 (5.90)							
	T3	17.89 (6.94)							
Reassure self	T1	18.00 (6.10)	*F*_(1, 25)_ = 7.32, ***p* = 0.007**, *η*_p_^2^ = 0.29	− 4.42	**0.003**	− 3.47	0.121	0.95	0.855
	T2	22.42 (5.06)							
	T3	21.47 (5.93)							
Hated self	T1	2.32 (.82)	*F*_(2, 36)_ = 2.52, *p* = 0.095, *η*_p_^2^ = 0.12	0.16	0.248	0.053	0.992	− 0.11	0.488
	T2	2.16 (.83)							
	T3	2.63 (.87)							
External shame	T1	54.37 (14.09)	*F*_(2, 27)_ = 2.94, *p* = 0.082, *η*_p_^2^ = 0.14	0.32	1.000	1.84	0.239	1.53	0.232
	T2	54.05 (13.09)							
	T3	52.53 (13.41)							
Social safeness and pleasure	T1	34.16 (8.01)	*F*_(1, 21)_ = 16.43, ***p* < 0.001**, *η*_p_^2^ = 0.48	− 7.37	**0.002**	− 7.47	**0.002**	− 0.12	1.000
	T2	41.53 (5.10)							
	T3	41.63 (5.91)							
Anxiety	T1	17.63 (3.89)	*F*_(1, 22)_ = 6.80, ***p* = 0.012**, *η*_p_^2^ = 0.27	0.90	0.051	1.84	**0.037**	− 0.053	1.000
	T2	15.74 (4.74)							
	T3	15.79 (4.88)							
Depression	T1	21.26 (6.05)	*F*_(2, 36)_ = 3.41, ***p* = 0.044**, *η*_p_^2^ = 0.16	0.53	0.140	0.63	0.126	0.11	1.000
	T2	20.74 (6.09)							
	T3	20.63 (6.30)							
Well-being	T1	45.63 (10.86)	*F*_(1, 20)_ = 11.12, ***p* = 0.002**, *η*_p_^2^ = 0.38	− 7.37	**0.009**	− 7.16	**0.010**	0.211	1.000
	T2	53.00 (6.57)							
	T3	52.79 (6.48)							

*MD* mean difference; *Sig.* significance level; *n*, number of participants

### Maintenance of Efficacy of the CMT at Follow-up: UK Sample

A repeated-measures ANOVA was used to investigate whether the efficacy of CMT observed for the UK sample was maintained at follow-up (T3) 2 weeks after completing the CMT. Results indicated that not only all the changes observed at T2 were maintained at T3, but also further improvements were observed at T3.

Results for each of the three flows of compassion indicated significant improvements across the three time points, with large effect sizes in self-compassion and compassion to others, and a medium effect size in compassion from others. Bonferroni corrected tests indicated that each of the three flows of compassion increased significantly from T1 to T2. Self-compassion also increased from T1 to T3, but not from T2 to T3. Similarly, all three types of fears of compassion also changed significantly across the three time points with large effect sizes in fear of compassion to others and fear of compassion from others, and a medium effect size in fear of self-compassion. Other inhibitors of compassion such as inadequate self, external shame, anxiety, and depression also changed significantly across the three time points with a medium effect in external shame and large effects in the other outcomes. Of the facilitators of compassion, social safeness and pleasure increased significantly with a large effect size, which was not reported at post CMT. Significant changes were observed from T1 to T2, T1 to T3, and T2 to T3 in the fear of compassion to others. Significant changes were only observed from T1 to T3 in the fear of compassion from others. For inadequate self, anxiety, and depression, significant changes were found from T1 to T2, and T1 to T3, but not from T2 to T3. A significant change was observed from T1 to T2, but not from T1 to T3 or T2 to T3 in the social safeness and pleasure scores (Table 10).

**Table 10:** Changes across time in the UK sample

Measure	Time	CMT group (*n*_T1_ = 125, *n*_T2_ = 73, *n*_T3_ = 36) *M (SD)*	Tests of within-subject effects	T1 vs T2		T1 vs T3		T2 vs T3	
			Time	*MD*	Sig	*MD*	Sig	*MD*	Sig
Self-compassion	T1	58.89 (12.66)	*F*_(2, 55)_ = 21.51, ***p* < 0.001**, *η*_p_^2^ = 0.38	− 9.31	** < 0.001**	− 9.53	** < 0.002**	− 0.22	1.000
	T2	68.19 (14.89)							
	T3	68.42 (15.94)							
Compassion to others	T1	76.73 (10.91)	*F*_(2, 72)_ = 9.08, ***p* < 0.001**, *η*_p_^2^ = 0.20	− 14.35	** < 0.001**	− 1.70	0.366	2.65	**0.021**
	T2	81.08 (10.23)							
	T3	78.43 (10.95)							
Compassion from others	T1	57.73 (15.96)	*F*_(2, 72)_ = 4.58, ***p* = 0.013**, *η*_p_^2^ = 0.11	− 6.22	**0.028**	− 5.37	0.109	0.84	1.000
	T2	63.95 (16.68)							
	T3	63.11 (16.33)							
Fear of compassion to others	T1	28.43 (7.43)	*F*_(2, 72)_ = 13.75, ***p* < 0.001**, *η*_p_^2^ = 0.28	− 3.14	**0.037**	5.43	** < 0.001**	2.30	**0.047**
	T2	35.30 (9.43)							
	T3	23.00 (8.13)							
Fear of compassion from others	T1	30.03 (8.86)	*F*_(1, 49)_ = 6.10, ***p* = 0.010**, *η*_p_^2^ = 0.15	2.97	0.129	4.14	**0.018**	1.16	0.289
	T2	27.05 (10.46)							
	T3	25.89 (19.99)							
Fear of self-compassion	T1	34.59 (11.82)	*F*_(1, 44)_ = 4.45, ***p* = 0.033**, *η*_p_^2^ = 0.11	4.19	0.083	4.14	0.129	− 0.05	1.000
	T2	30.41 (14.56)							
	T3	30.46 (14.36)							
Inadequate self	T1	22.05 (8.56)	*F*_(1, 52)_ = 13.38, ***p* < 0.001**, *η*_p_^2^ = 0.27	4.47	**0.003**	5.18	** < 0.001**	0.71	0.815
	T2	17.58 (8.83)							
	T3	16.87 (8.83)							
Reassure self	T1	15.97 (7.02)	*F*_(2, 59)_ = 0.09, *p* = 0.885, *η*_p_^2^ = 0.00	0.03	1.000	0.19	1.000	0.17	1.000
	T2	15.94 (6.21)							
	T3	15.78 (5.11)							
Hated self	T1	1.92 (0.80)	*F*_(2, 72)_ = 2.70, *p* = 0.074, *η*_p_^2^ = 0.07	− 0.03	1.000	0.14	0.173	0.16	0.170
	T2	1.95 (0.85)							
	T3	1.78 (0.82)							
External shame	T1	45.00 (14.07)	*F*_(1, 54)_ = 3.75, ***p* = 0.042**, *η*_p_^2^ = 0.09	4.41	0.077	4.73	0.164	0.32	1.000
	T2	40.59 (15.53)							
	T3	40.27 (16.19)							
Social safeness and pleasure	T1	35.30 (9.01)	*F*_(2, 61)_ = 7.66, ***p* = 0.002**, η_p_^2^ = 0.18	− 4.49	**0.002**	− 3.00	0.094	1.49	0.328
	T2	39.78 (9.76)							
	T3	38.30 (10.38)							
Anxiety	T1	17.19 (6.35)	*F*_(2, 57)_ = 8.13, ***p* = 0.002**, η_p_^2^ = 0.18	2.38	**0.018**	2.76	**0.007**	0.38	1.000
	T2	14.81 (5.69)							
	T3	14.43 (5.44)							
Depression	T1	20.05 (6.55)	*F*_(1, 52)_ = 7.45, ***p* = 0.004**, *η*_p_^2^ = 0.17	2.73	**0.028**	2.81	**0.009**	0.08	1.000
	T2	17.32 (5.64)							
	T3	16.24 (5.70)							
Well-being	T1	42.97 (9.79)	*F*_(2, 72)_ = 2.79, *p* = 0.068, *η*_p_^2^ = 0.07	− 2.89	0.142	− 2.92	0.256	− 0.03	1.000
	T2	45.86 (11.19)							
	T3	45.89 (11.23)							

*MD* mean difference; *Sig.* significance level; *n*, number of participants

### Feedback on the CMT Engagement

Most of the participants in both countries reported that they were able to engage in the CMT practices “quite well” (ranging from 40 to 46.22%) or “very well’ (ranging from 22.68 to 33.57%) every day, across the 14-day period of the intervention.

In addition, most participants from both countries “strongly agreed*”* that the CMT was helpful (63% SL, 39% UK), accessible (57% SL, 48% UK), and feasible (52% SL, 44% UK), implying that participants may have had a positive experience from engaging in the CMT. One distinction was that majority of the UK participants only “slightly agreed” that they were able maintain their compassionate self every day (29.9%) post CMT, although a majority of the UK participants “strongly agreed” (40.2%) that they were able to maintain a compassionate self at follow-up. Other than that, most participants from both countries “strongly agreed” that they would have liked to continue the CMT (50% SL, 39% UK), would like to use the CMT in future (35% SL, 42% UK), and continue to feel its benefits at a 2-week follow-up (52% SL, 42% UK). However, only a few participants from both countries completed the post-intervention (*n* = 37 Sri Lankan, and *n* = 88 from UK) and follow-up (*n* = 19 Sri Lankan, and *n* = 36 UK) feedback questions.

## Discussion

This study explored the efficacy of a brief 2-week online CMT, in a non-clinical, cross-cultural group of Sri Lankan and UK participants. The CMT significantly increased compassion across the three flows regardless of their cultural background. Significant improvements were indicated in the facilitators (e.g., self-reassurance, social safeness and pleasure, and well-being) and in the inhibitors of compassion (fears of compassion, self-inadequacy, external shame, anxiety, and depression) with some cross-cultural differences. The results found that not only were all post-CMT changes maintained, but also further improvements were observed in some variables. Although there was a high attrition rate, participants who completed the feedback questions indicated that they found the CMT useful and accessible, and that they would recommend the CMT to others. This suggests that the CMT is a feasible practice for the public, which was also demonstrated in previous cross-cultural studies (Maratos et al., 2020; Matos et al., 2021, 2022a, 2022b).

Significant improvements in self-compassion, compassion to others, and compassion from others were reported in the Sri Lankan CMT group immediately post CMT, with large effect sizes (in the PP analysis). Significant improvements in self-compassion, compassion to others, and compassion from others were also reported in the UK CMT group immediately post CMT. However, only self-compassion in the UK group increased with a large effect size, and compassion to and from others increased with small effect sizes.

A possible explanation for the differences in effect sizes between the Sri Lankan and UK CMT groups is that, while there was no significant difference in self-compassion between the two countries at baseline-1, UK participants indicated significantly higher levels of compassion to and from others prior to starting the CMT. A similar UK CMT study found significant increases in self-compassion, but not in compassion towards and from others, and emphasized that many participants described being already “too compassionate” prior to the CMT and, therefore, the CMT was mostly effective in increasing self-compassion (Irons & Heriot-Maitland, 2021). In addition, while the CMT includes practices to improve all three flows of compassion, the focus is weighted towards improving self-compassion (Irons & Heriot-Maitland, 2021), which may be why compassion to or from others did not improve with large effect sizes in the UK group who already had higher scores prior to CMT. Despite the different effects, results are in line with previous studies (Irons & Heriot-Maitland, 2021; Maratos et al., 2020; Matos et al., 2017a), that the CMT can improve people’s compassion for themselves and others in not just Western cultures, but Eastern cultures as well. Results were also maintained at a 2-week follow-up, supporting the lasting effects of the CMT (Gilbert & Procter, 2006; Irons & Heriot-Maitland, 2021).

Significant increases in self-reassurance, social safeness and pleasure, and well-being were reported immediately post CMT in the Sri Lankan CMT group. In addition, fear of compassion from others, fear of self-compassion, and inadequate-self were significantly decreased. However, fear of compassion to others, hated-self, anxiety, or depression did not decrease post CMT. While results were maintained at 2-week follow-up, anxiety and depression significantly decreased, which were not reported immediately post CMT. In the UK CMT group, fear of compassion to others, fear of compassion from others, fear of self-compassion, inadequate-self, external shame, anxiety, and depression significantly decreased post CMT. Results were maintained at follow-up, while social safeness and pleasure significantly increased at follow-up, which was not reported immediately post CMT. Although compassion across the three flows increased after the CMT, significant improvements were not observed in the facilitators of compassion such as self-reassurance, social safeness and pleasure, or well-being in the UK CMT group, which is in direct contrast to the Sri Lankan participants. This finding directly contradicts previous CMT studies that found increases in self-reassurance (Gilbert & Procter, 2006; Irons & Heriot-Maitland, 2021) and well-being (Irons & Heriot-Maitland, 2021) in UK people. This, however, is not an indication that the CMT was not as effective in the UK sample, as the inhibitors (e.g., fears, shame, depression, anxiety) reduced and social safeness increased later at the follow-up in the UK people.

Self-reassurance in the Sri Lankan participants was significantly higher compared to UK participants at baseline-1 (T1), which highlights the presence of subtle cultural differences. This was also replicated in a previous study where self-reassurance was greater in Sri Lankans compared to UK people, which was attributed to the cultural difference such as the strong Buddhist influence in Sri Lanka (Kariyawasam et al., 2022a). This could be a possible reason for the increased self-reassurance post CMT in this study, given that the majority of the Sri Lankan participants were Buddhists (51.7%) and the CMT included practices similar to Buddhist meditation (e.g., mindfulness practices). This could also partially explain the lack of significant change in self-reassurance in the UK group as only 2% of the UK group were Buddhists.

Self-inadequacy decreased in both Sri Lankan and UK groups post CMT. This was expected for the Sri Lankans as their self-reassurance, the opposite of self-inadequacy (Gilbert, 2014), significantly increased. While this is in line with previous CMT studies (Irons & Heriot-Maitland, 2021; Matos et al., 2022a), it is an important finding, as increased self-reassurance and decreased self-criticism have indicated decreased psychopathology and increased well-being (Irons & Heriot-Maitland, 2021). This was also evident in the present study post CMT (well-being increased in the Sri Lankans and depression and anxiety decreased in the UK group). On the other hand, UK participants’ decreased self-inadequacy suggests that although their self-reassurance did not increase significantly, their self-criticism may have decreased, which is also in line with a previous CMT conducted in a UK sample (Irons & Heriot-Maitland, 2021). Moreover, decreases in self-inadequacy are comprehensible given that self-inadequacy is negatively associated with self-compassion and both Sri Lankan and UK participants’ self-compassion significantly increased (Gilbert et al., 2014).

Significant increases in well-being, social safeness, and pleasure scores were also observed in the Sri Lankan participants, post CMT, which is in line with the emphasis by Irons and Heriot-Maitland (2021), that CMT practices are effective not only in reducing distress and mental illness, but also in increasing positive affect and well-being. In fact, the goal of CMT is to facilitate people’s well-being by promoting compassion for the self and others, and by decreasing distress and psychopathology (Gilbert, 2020). Supporting this, several CMT studies have reported increases in well-being (Kirby et al., 2017).

The increased social safeness and pleasure add a valuable contribution to the impact of CMT in collectivistic societies such as Sri Lanka, as previous studies indicate that people in such cultures can feel insecure in their social relationships and feel that they are constantly judged by their society (Kariyawasam et al., 2021; Van-Hoorn, 2014). Social shame and criticism are encouraged in such cultures to reflect on one’s shortcomings and failures, as this can motivate people to not repeat mistakes (Abeyasekara & Marecek, 2019; Kitayama & Uchida, 2003). This, however, may increase people’s fears towards others and can be associated with a feeling of lack of warmth and safeness. There is lack of robust research to support these very plausible and logical explanations, as there are other variables in place that may impact on feelings of warmth and safeness in these cultures (Gilbert et al., 2014; Kariyawasam et al., 2021, 2022a). Studies have discussed how Sri Lankan people perceive their society to be the biggest inhibitor of their compassionate experiences, and that they are significantly fearful of compassion and feel less social safeness, when compared to UK people (Kariyawasam et al., 2021, 2022a). This is in line with the present study as significantly higher levels of fears of compassion across the three flows of compassion were reported in the Sri Lankan group at baseline-1 (prior to starting CMT). Therefore, the increased social safeness and pleasure scores in the present study in the CMT group indicate that the CMT may potentially have been helpful in reducing the barrier of social insecurities faced by Sri Lankan participants.

Although social safeness and pleasure increased post CMT, external shame, which was significantly higher in the Sri Lankans compared to UK participants at baseline-1, did not significantly decrease post CMT. It is important to understand that some Asian collectivistic cultures consider shame as a valuable concept towards perfection and believe that social shame guides people to correct their mistakes (Geaney, 2004; Neff et al., 2008). Although external shame in the present study was considered an inhibitor of compassion, it is possible that Sri Lankan participants may have perceived it as an important indicator of well-being as social shame is embedded in the Sri Lankan culture (Abeyasekera & Marecek, 2019). In addition, people in Asian collectivistic cultures turn only to their family and closest friends when seeking social support, whereas people in Western individualistic cultures refer to a broader circle of family, long-term and recent friends, and acquaintances when seeking social support (Huang, 1994; Perez, 1997). While the Sri Lankan group may have referred to the society in general when reporting their perceived external shame, there is a possibility that they only considered family and closest friends when reporting social safeness. Shame experiences have shown associations with the activation of the threat system, which may cause heightened fears and negative self-evaluations, and the underdevelopment of the soothing system, which may cause negative perceptions that others are not safe and trustworthy (Gilbert, 2009; Matos et al., 2015). In line with this, despite completing the CMT, Sri Lankan participants’ external shame or fear of compassion towards others remained unchanged. In contrast, significant decreases were reported for external shame in the UK participants post CMT, which was reflected in their lack of fear of compassion towards and from others indicating that results in the present study are in line with previous UK studies (Gilbert & Procter, 2006).

Anxiety in the UK group was higher compared to that in the Sri Lankan group at baseline-1, and both depression and anxiety scores in the UK participants significantly reduced post CMT. In partial support, studies found that CMT reduced depression but not anxiety in non-clinical populations (Irons & Heriot-Maitland, 2021) and both depression and anxiety reduced in clinical populations. In fact, CMT has found to be more effective in depression than CBT, which specifically targets reducing depression (Kelman et al., 2018). All post-CMT improvements were maintained at a 2-week follow-up in both countries further supporting the lasting effects of the CMT (Gilbert & Procter, 2006; Irons & Heriot-Maitland, 2021). Another clinically relevant finding is that anxiety and depression reduced in the Sri Lankan group, and social safeness and pleasure increased in the UK group at follow-up, which is particularly noteworthy given that these emotions did not improve immediately post CMT. Additionally, compassion to others and fear of compassion to others in the UK group improved further at follow-up. This not only highlights the potential of CMT to have a positive influence of these emotions in the longer term but also that the positive effect may be delayed. In fact, most existing CMT studies have explored the long-term effects rather than short-term effects (Zessin et al., 2015). Irons and Heriot-Maitland (2021) stated that improvements observed post CMT were not only sustained, but also continued to improve, although they emphasized that improvements need to be carefully interpreted, as there is a possibility that people who completed the follow-up are more likely to be participants with a high enthusiasm for the CMT.

This study provided promising evidence for the cross-cultural applicability and effectiveness of CMT, particularly in Sri Lanka communities, which has been predominantly applied in Western settings. The cross-cultural differences and religion should be accounted when tailoring interventions and treatments. For instance, external shame was higher in Sri Lankan participants, and anxiety was higher in UK participants prior to the CMT. Therefore, a CMT aimed at Sri Lankans could incorporate more practices to reduce the impact of social shame (e.g., by adding friendly voice tones, imagery tasks of compassionate others), while a CMT aimed for UK people could add practices to minimize levels of anxiety (e.g., soothing rhythm breathing). Clinicians should closely look at baseline assessment results to understand which practices are needed to balance the affective regulatory system. For instance, if both fears and social safeness are significantly higher at baseline, this may be an indicator that activities to reduce the threat system (e.g., mindfulness) should be prioritized over activities to induce the soothing system, for that specific sample. Prospective studies should also consider taking a mixed-method approach by incorporating qualitative interviews, to better understand the feasibility of CMT and challenges faced, and to explore possible reasons for the large attrition rates (Maratos et al., 2019).

### Limitations and Directions for Future Research

This study used a RCT design, together with a series of validated questionnaires to measure a variety of aspects of compassion using an online CMT as a potentially effective mode of delivery. The use of an already established CMT, which has been recognized to be effective in Western communities (Matos et al., 2017a), was an advantage of this study. The results suggest that although CMT was developed as a group-based therapy (Gilbert & Irons, 2004), the intervention was accessible to a larger non-clinical sample and has the potential to be as effective as an in-person CMT. The incorporation of feedback questionnaire further contributed to the methodological rigor of the study.

One of the biggest limitations of this study is the high attrition rate. High attrition is a common concern among many online interventions (Eysenbach, 2005) and was expected considering the level of commitment required for the intervention (Halamova et al., 2020). More importantly, the high attrition rate was expected as data collection took place during the COVID-19 period in both countries, and during a political and economic crisis in Sri Lanka (Al-Jazeera, 2022; World Bank, 2022). It is possible that only those with an interest in the intervention may have completed the study (Halamova et al., 2020). The large dropout rate also resulted in the small sample size and was a reason for conducting intention-to-treat analyses to understand the intervention efficacy (Arimitsu, 2016).

Although the use of a series of validated measures strengthened the understanding of compassion and its association with inhibitors and facilitators of compassion, this may have increased the common method bias. It is the variance caused by the use of self-report measures that measure multiple constructs (e.g., the CEAS measures all three flows of compassion, the FSCRC measures self-inadequacy, self-hatred, and self-reassurance), as they are measured using the same method (Podsakoff & Organ, 1986). This is because participants having to report their perceptions on two or more constructs in the same scale is likely to produce spurious effects due to the scales used to measure the construct rather than the construct that is being measured (Podsakoff et al., 2012).

Significant decreases in all three flows of fears of compassion (fear of compassion to and from others, fear of self-compassion) in the UK CMT group imply that the CMT not only increases compassion across the three flows, but also has the capacity to reduce the fears associated with these flows. It is important for future research to explore the direction of its functionality (e.g., whether the reduction in fears of compassion leads to an increase in compassion, or vice versa, or there may be another mechanism that facilitates these changes). In line with the findings, an American CFT study reported that the intervention significantly reduced all three flows of the fears of compassion while increasing self-compassion and compassion from others (Fox et al., 2021).

In line with previous research (e.g., Matos et al., 2017a, 2022a, 2022b), this study incorporated self-reported measures that directly accessed elements addressed by the CMT. This may have increased the risk of potential demand characteristics upon participants (Matos et al., 2022a), in addition to the increased social desirability effect (Guan et al., 2021). It was beyond the scope of this study to go into detail about the impact of each component of the CMT, such as psychoeducation, imagery, and breathing, which Matos et al. (2017a) demonstrated would be useful for future studies to address. Additionally, this study cannot be generalized to clinical populations; and thus, future studies should replicate this study with a clinical sample to investigate the accessibility and feasibility, and effectiveness of an online CMT in clinical samples towards reducing psychopathology (Halamova et al., 2020).

## Data Availability

Data relating to this study can be found from the University of Southampton Institutional Repository upon request (https://eprints.soton.ac.uk/472085/).
